# Percepciones de profesionales del servicio de pediatría sobre la intervención “Consejo breve anti-alcohol en edad pediátrica” en Asturias, España

**DOI:** 10.18294/sc.2024.4963

**Published:** 2024-10-22

**Authors:** Ángel Alonso-Domínguez, Sonia Otero-Estévez, Rocío Pérez-Gañán

**Affiliations:** 1 Doctor en Sociología. Profesor e investigador, Departamento de Sociología, Universidad de Oviedo. Miembro del grupo de investigación consolidado Promoviendo el Empleo y el Bienestar en Europa (PROMEBI), Gijón, España. alonsodangel@uniovi.es Universidad de Oviedo Departamento de Sociología Universidad de Oviedo Gijón Spain alonsodangel@uniovi.es; Grupo de investigación consolidado Promoviendo el Empleo y el Bienestar en Europa (PROMEBI); 2 Doctora en Sociología. Profesora e investigadora, Departamento de Sociología, Universidad de Oviedo. Miembro del grupo de investigación consolidado de Sociología de la Alimentación (SOCIALIMEN), Oviedo, España. oterosonia@uniovi.es Universidad de Oviedo Departamento de Sociología Universidad de Oviedo Oviedo Spain oterosonia@uniovi.es; Grupo de investigación consolidado de Sociología de la Alimentación (SOCIALIMEN) Gijón; 3 Doctora en Antropología Social y Cultural. Profesora e investigadora, Departamento de Sociología, Universidad de Oviedo. Miembro del grupo de investigación consolidado de Sociología de la Alimentación (SOCIALIMEN), Gijón, Españasicóloga Clínica. Estudiante Programa de doctorado en estudios interdisciplinares de género, Universidad Rey Juan Carlos, Madrid, España. perezganmaria@uniovi.es Universidad Rey Juan Carlos Universidad Rey Juan Carlos Madrid Spain perezganmaria@uniovi.es; Grupo de investigación consolidado de Sociología de la Alimentación (SOCIALIMEN) Gijón; Departamento de Sociología Universidad de Oviedo Oviedo

**Keywords:** Sociología, Consumo de Alcohol en Menores, Evaluación de Procesos, Atención de Salud, España, Sociology, Underage Drinking, Process Assessment, Health Care, Spain

## Abstract

El objetivo de este estudio, fundamentado en las teorías del cuidado de la salud y del aprendizaje social, es conocer el grado de implementación y alcance del protocolo del “Consejo breve anti-alcohol en edad pediátrica”, una intervención preventiva sobre el consumo de alcohol en menores del Servicio de Salud del Principado de Asturias, España. A partir de una metodología cualitativa, basada en trece entrevistas semiestructuradas realizadas en junio de 2022, se analiza el desarrollo del protocolo, las percepciones de profesionales del servicio de pediatría sobre su implementación, y sus representaciones culturales sobre el consumo de alcohol en el contexto asturiano y español. Los resultados revelan un alcance limitado debido a la heterogeneidad en su aplicación y la dificultad de medir su impacto, comparado con otras áreas de la salud pediátrica. Asimismo, se subraya la importancia de la dimensión educadora y cuidadora de las áreas de pediatría y enfermería, y la necesidad de un enfoque integral que involucre a las familias y otras instituciones para mejorar la prevención del consumo de alcohol desde edades tempranas.

## Abstract (en)

The aim of this study, based on health care and social learning theories, is to understand the degree of implementation and reach of the “Brief Anti-Alcohol Counseling in Pediatric Age” protocol, a preventive intervention addressing alcohol consumption among minors within the Health Service of the Principality of Asturias, Spain. Using a qualitative methodology, based on thirteen semi-structured interviews conducted in June 2022, the study analyzes the development of the protocol, the perceptions of pediatric service professionals regarding its implementation, and their cultural representations of alcohol consumption within the Asturian and Spanish context. The results reveal limited reach due to the heterogeneity in its application and the difficulty in measuring its impact compared to other areas of pediatric health. Furthermore, the importance of the educational and caregiving dimensions of the pediatric and nursing areas is emphasized, as well as the need for a comprehensive approach that involves families and other institutions to improve alcohol consumption prevention from an early age.

## INTRODUCCIÓN

El consumo de alcohol en menores es un problema de gran relevancia en la salud pública, por su prevalencia y por las graves consecuencias que conlleva. Diversos estudios han demostrado que la iniciación temprana en el consumo de alcohol aumenta de forma significativa la probabilidad de desarrollar conductas de riesgo y adicciones en la edad adulta^1^^,^^2^. A pesar de ello, tanto la literatura científica como los datos que ofrecen los organismos internacionales muestran que el alcohol es la sustancia de mayor consumo en edad pediátrica^3^, revelando algunos patrones característicos de su ingesta, como la intensidad o “atracones”^4^^)^ y la baja edad de inicio al consumo, ligeramente inferior a los catorce años^1^^,^^2^. Según un estudio global, el 25% de los adolescentes de 12 a 15 años en países de ingresos bajos y medios han consumido alcohol al menos una vez en los últimos 30 días, con una prevalencia mayor en los niños de 14 y 15 años en comparación con los de 12 y 13 años^5^. En Europa, la Organización Mundial de la Salud (WHO, por sus siglas en inglés) reporta que las y los jóvenes de 15 a 19 años tienen las tasas más altas de consumo de alcohol^6^. La realidad no es muy diferente en España. Según el Observatorio Español de las Drogas y las Adicciones (OEDA), el 77,9% de los estudiantes de secundaria ha consumido alcohol alguna vez, y el 32,3% ha participado en episodios de consumo intensivo en el último mes^7^. Investigaciones recientes subrayan que las y los adolescentes españoles presentan una mayor prevalencia de conductas de riesgo relacionadas con el consumo de alcohol en comparación con otros países europeos^8^, evidenciando una problemática que combina factores individuales, sociales y culturales^9^^,^^10^^,^^11^^,^^12^. Estos datos revelan una transición del patrón de consumo mediterráneo, caracterizado por un consumo diario de bajo riesgo en el ámbito familiar, hacia un modelo nórdico, que se distingue por ingestas masivas en espacios públicos durante el fin de semana y abstinencia durante el resto de la semana. Las repercusiones sociales negativas asociadas a este cambio incluyen una alta mortalidad, agresiones, violencia doméstica, conflictos familiares y sociales, fracaso académico y, consecuentemente, un mayor abandono escolar^8^.

En el Estado español, la ingesta de alcohol está profundamente normalizada en el contexto social y familiar, lo que dificulta el reconocimiento de su consumo como un problema. La normalización del consumo, exacerbada por la baja percepción del riesgo y las escasas restricciones en la venta de alcohol a menores, así como el incumplimiento de las normativas existentes, aumenta su exposición a problemáticas derivadas de dicho consumo. 

Esta cultura del consumo de alcohol está, asimismo, muy arraigada en el Principado de Asturias^9^^,^^13^. Para tratar de dar respuesta a esta problemática en esta comunidad autónoma, en línea con otras iniciativas internacionales^14^^,^^15^, la Dirección General de Salud Pública (DGSP) y el Servicio de Salud del Principado de Asturias (SSPA) implementaron en 2016 el “Consejo breve anti-alcohol”, una intervención preventiva para abordar los daños y riesgos bio-psico-sociales propios del consumo de bebidas alcohólicas. El protocolo y la Guía que lo acompaña son una adaptación del *Alcohol Screening and Brief for Youth: a Practtitioner´s Guide* desarrollada por el National Institute on Alcohol Abuse and Alcoholism^16^ (NIAAA) de EEUU y constituye una medida de política social sanitaria pionera en España, con el objetivo de evitar la ingesta de alcohol a edades tempranas a través de consultas de pediatría. 

En relación con el protocolo de intervención del Consejo breve anti-alcohol, la evaluación del riesgo comienza con dos preguntas de cribado que deben realizarse en el orden adecuado. Se formula, en primer lugar, una pregunta referida al consumo del grupo de amigas y amigos y, ante cualquier consumo, se anota en el software para centros de atención primaria OMI-AP. A continuación, se pregunta si ha consumido alcohol en alguna ocasión. Si la respuesta es afirmativa, sea cual fuere la pauta de consumo, se anota como riesgo alto. En el siguiente paso, a las y los jóvenes que no beben ni tampoco lo hace su grupo de iguales, se les elogian esos hábitos saludables. Además, a las personas abiertas a recibir más información se les refuerzan las conductas, exponiendo los riesgos físicos, psicológicos y sociales a los que se exponen con el consumo de alcohol. Al mismo tiempo, se tratan de averiguar las razones para mantenerse alejadas del alcohol y la forma en la que piensan permanecer al margen. Finalmente, a quienes han declarado consumir alcohol, se las clasifica en riesgo alto, moderado o bajo, en función de una serie de parámetros vinculados al tiempo de exposición en el último año, la edad y la existencia o no de personas bebedoras en su círculo de amistades. El protocolo finaliza con el asesoramiento y la ayuda necesarias que les permitan a las y los jóvenes establecer un objetivo y un plan personal de acción.

En la intervención del Consejo breve anti-alcohol se considera que las y los profesionales sanitarios son la figura ideal para acercar las recomendaciones a las y los jóvenes que utilizan los servicios de pediatría, ya que los considerarían como la mejor vía de información, muy por delante de familias, profesorado, medios de comunicación o grupo de pares. El acceso al programa es universal, ya que las preguntas protocolizadas se realizan en todas las consultas del “niño sano” en dos momentos temporales distintos: la revisión de los diez años, en primera instancia y la de los trece años, en segunda y última, ya que esta edad marca la frontera límite de la pediatría en el Principado de Asturias.

En el ámbito de la investigación, las intervenciones breves han mostrado una notable efectividad en la reducción de las tasas de consumo de sustancias y problemas relacionados. Estas intervenciones se centran en la identificación temprana de individuos que consumen alcohol y otras drogas, proporcionando un programa antes de que los sujetos lo soliciten o sean conscientes de que su patrón de consumo podría causarles problemas. Las intervenciones breves desde pediatría para la prevención del consumo de alcohol se pueden enmarcar dentro de la noción de “cuidado” que subyace en los modelos contemporáneos de atención sanitaria. Esta perspectiva no solo se enfoca en la intervención directa sobre el comportamiento de las y los adolescentes, sino que también integra una visión más amplia que abarca el contexto social, emocional y familiar de la persona. Desde esta óptica, el personal de pediatría no solo actúa como proveedor de salud, sino también como cuidador y educador, encargado de guiar y apoyar a las y los menores en su desarrollo, al tiempo que se fortalece el papel de la familia en la promoción de hábitos saludables^17^^,^^18^^,^^19^. Asimismo, en su dimensión práctica, se apoya en la teoría del aprendizaje social^20^, de la que se deriva que el consumo de alcohol y otras drogas se adquiere a través de la exposición y experimentación de patrones de comportamiento. Estos patrones pueden modificarse mediante nuevas situaciones de aprendizaje, en la que los individuos son el componente activo en el proceso de cambio de su consumo y en la implementación de estrategias para controlar su consumo y evitar recaídas^21^^,^^22^. Aunque la aplicación de las intervenciones breves ha sido mayormente en adultos, se están realizando esfuerzos para adaptar este modelo a adolescentes que abusan de sustancias, pero que aún no presentan dependencia, prestando una especial atención a la intervención en los entornos escolares^22^ y en el contexto clínico, donde las intervenciones breves administradas por personal de enfermería han mostrado cierto éxito en la reducción del consumo de alcohol entre adolescentes^23^. 

Sin embargo, la eficacia de estas intervenciones ha sido percibida como limitada debido a diversos obstáculos y a la heterogeneidad en su aplicación. Además, las intervenciones breves con menores y su evaluación, aún son escasas y no presentan resultados concluyentes^24^^,^^25^, lo que subraya la importancia, por un lado, de desarrollar programas de intervención breve como una alternativa para adolescentes que comienzan a abusar del alcohol u otras drogas y, por otro, de evaluar la implementación de estas intervenciones breves, para plantear mejoras en su eficacia. En este contexto, el presente trabajo se erige como necesario para comprender las percepciones y experiencias de las y los profesionales del servicio de pediatría en la implementación del Consejo breve anti-alcohol, proporcionando información valiosa para mejorar la eficacia de esta intervención preventiva y desarrollar estrategias más efectivas para abordar el consumo de alcohol en menores en el Principado de Asturias.

Por lo anteriormente expuesto, la presente investigación ha tenido como objetivo principal conocer el grado de implementación y alcance del protocolo del Consejo breve anti-alcohol realizada con menores en edad pediátrica en el Principado de Asturias. Este objetivo principal se ha articulado en tres objetivos específicos: 1) analizar el desarrollo y la implementación del protocolo del Consejo breve anti-alcohol en menores por parte del personal de atención primaria; 2) conocer las percepciones de las y los profesionales del servicio de pediatría sobre la efectividad y los desafíos de la intervención preventiva anti-alcohol; y 3) indagar sobre las representaciones culturales y creencias de las y los profesionales sobre el consumo de alcohol en menores en el contexto asturiano y español. 

### Perspectivas de análisis sobre las intervenciones breves en la prevención del consumo de alcohol en menores

En la actualidad, desde la producción científica especializada, existe un consenso generalizado de que la mejor forma de combatir la iniciación en el consumo de drogas y las adicciones es la prevención temprana y las intervenciones continuadas, entre las que se encuentran estrategias como el cribado en la atención primaria de la salud y los consejos breves^7^^,^^26^^,^^27^^,^^28^^,^^29^^,^^30^. En este sentido, las intervenciones breves se alinean con la noción de cuidado integral, al proporcionar un enfoque personalizado que aborda no solo los comportamientos de riesgo, como el consumo de alcohol, sino también los factores subyacentes que pueden influir en estos comportamientos, tales como las dinámicas familiares, las presiones sociales y la autoestima del adolescente^31^. Este enfoque integral es esencial para la efectividad de las intervenciones, ya que considera al individuo en su totalidad, promoviendo un entorno de apoyo que refuerza tanto la salud física como el bienestar emocional. Las intervenciones breves también reflejan la importancia de la prevención y la promoción de la salud dentro del cuidado pediátrico. Estas intervenciones permiten la identificación temprana de conductas de riesgo y la implementación de estrategias preventivas antes de que el consumo de alcohol se convierta en un problema mayor. Asimismo, este enfoque preventivo es coherente con los modelos de atención que buscan no solo tratar la enfermedad, sino también evitar su aparición mediante la promoción de estilos de vida saludables y el fortalecimiento de la resiliencia individual y familiar.

 La bibliografía sobre la cuestión refleja de manera profusa que las y los profesionales del servicio de pediatría de atención primaria son conscientes del problema creciente que constituye el alcohol y también de que el consumo de esta sustancia es un hábito social muy arraigado. Al mismo tiempo, también se considera que han adquirido una posición estratégica tanto en la prevención primaria, basada en la educación sanitaria, como en la detección precoz, que constituiría la prevención secundaria y la colaboración con otras instancias sanitarias propia de la prevención terciaria^7^. Así, el rol de las y los profesionales del servicio de pediatría en este contexto es fundamental. Como personas cuidadoras y educadoras, el personal de pediatría tiene la responsabilidad de guiar a las y los adolescentes y sus familias a través de las decisiones críticas relacionadas con la salud. Las intervenciones breves se convierten así en una herramienta esencial para que las y los profesionales del servicio de pediatría ejerzan esta función. Además, estas intervenciones refuerzan la noción de empoderamiento en el cuidado, al involucrar activamente tanto a las y los adolescentes como a su familia en el proceso de toma de decisiones, lo que aumenta la probabilidad de éxito de la intervención a largo plazo^32^. 

Sin embargo, la evidencia científica también ha puesto de relieve la existencia de diversas barreras que dificultan la intervención de profesionales sanitarios, tales como la escasez de tiempo y la ausencia o la inadaptación de la formación que reciben, lo cual redunda en escasez de herramientas e insuficientes habilidades para abordar las situaciones y facilitar consejos útiles para la prevención temprana^26^^,^^30^.

Otra cuestión que señalan las investigaciones son los problemas a los que se enfrentan las y los profesionales sanitarios por la ausencia de empatía por parte de las familias y la oposición que, en ocasiones, encuentran en ellas para poder desarrollar los protocolos de manera adecuada, por ejemplo, durante la realización de una entrevista a solas con pacientes en edad pediátrica^30^. A ello habría que añadir ciertos sesgos cognitivos de las y los profesionales sanitarios, como factores discriminantes en relación con las actividades preventivas ante el consumo excesivo de alcohol. Algunas investigaciones mencionan, además, la existencia de un porcentaje elevado de profesionales sanitarios (donde el sexo y la edad son variables a tener en cuenta) que consumen a diario o casi a diario alcohol por gusto o costumbre. Esta ambigüedad y ambivalencia podría reflejarse en la no aplicación correcta de cribados y protocolos, en la invitación a un consumo moderado, en la renuncia a investigar en consulta el abuso del alcohol y en el escaso optimismo acerca de la falta de eficacia de medidas preventivas a temprana edad^7^^,^^33^^,^^34^.

La puesta en marcha del Consejo breve anti-alcohol en la consulta de pediatría de atención primaria de Asturias, a los 10 y a los 13 años, se fundamenta en esa exposición al riesgo, conductual y actitudinal del consumo de alcohol, que aumenta para quienes comienzan antes su consumo. En la literatura especializada se destaca una mayor prevalencia de conductas de riesgo respecto a la ingesta de alcohol entre adolescentes en España, en comparación con el promedio europeo y de otros países desarrollados^9^^,^^10^^,^^11^^,^^30^^,^^35^.

Las investigaciones realizadas en Asturias corroboran los resultados de otros estudios que se han realizado a nivel nacional y explican la problemática a partir de factores individuales y ambientales. Por una parte, las escasas restricciones a la venta de alcohol a menores y/o el incumplimiento de la norma^9^, así como la baja percepción de riesgo por consumo excesivo de alcohol, ya que no existe ningún tipo de síntomas al inicio, lo que implica, a su vez, que las personas más jóvenes solo acuden a los servicios sanitarios de urgencia cuando ya es visible el abuso y/o la dependencia^12^^,^^13^. Es importante señalar que este es un problema especialmente relevante entre los varones y, en este sentido, la literatura recoge que mujeres y hombres podrían tener, en relación con el consumo de alcohol, actitudes, valores y conductas diferentes^9^^,^^10^^,^^11^^,^^35^.

## METODOLOGÍA

En este estudio se ha realizado una evaluación del proceso de implementación y alcance de la intervención desde las percepciones de las y los profesionales encargados de la aplicación del Consejo breve anti-alcohol. Es por ello que, en consonancia con los objetivos planteados, se ha adoptado un enfoque de carácter cualitativo^36^, que permite explorar la interacción entre la intervención y el contexto en el que esta se produce y la utilización interrelacionada de diferentes técnicas de recogida de datos. En este caso, la principal técnica utilizada fue la entrevista semi-estructurada, debido a su gran potencial para hacer emerger los motivos y razones (discursos) que se encuentran detrás de las prácticas, así como para la obtención de un conocimiento profundo sobre el fenómeno a estudiar^37^^,^^38^^,^^39^.

De acuerdo con el planteamiento descrito, el proceso de selección de la muestra se llevó a cabo conforme con tres criterios considerados metodológicamente relevantes: el área sanitaria donde se desarrolla la actividad, el sexo, y la edad del personal sanitario que ha de proporcionar el consejo breve. Con base en la bibliografía, se consideró que estas tres variables podían plantear actitudes y conductas diferentes con relación al consumo de alcohol en menores y a la mayor o menor aplicación del consejo breve anti-alcohol propiciado por sesgos cognitivos de carácter individual y contextual. 

Como resultado de la combinación de los tres criterios expuestos se diseñó una muestra compuesta por dieciséis perfiles de entrevistas que, inicialmente, estaban contempladas solo con profesionales de pediatría, ya que desde la Consejería se entendía que eran quienes se encargaban de dar el consejo breve en los diversos centros de salud. Sin embargo, la perspectiva circular y abierta que permite el análisis cualitativo mostró la conveniencia de dialogar también con profesionales de enfermería, ya que en la fase de contacto se descubrió que, en muchas ocasiones, eran las personas que en la práctica se encargaban de realizar el protocolo del Consejo breve anti-alcohol. Este listado se acompañó también de un número de personas sustitutas, para prevenir posibles incidentes que impidieran la realización de las entrevistas a las y los profesionales seleccionados en primera instancia. Este diseño muestral no pretendía en modo alguno la obtención de una representatividad estadística, ya que este no es el propósito de la metodología cualitativa, sino dar cuenta y recoger la heterogeneidad de discursos y acciones relacionadas con el Consejo breve anti-alcohol, dependiendo de la posición social que se ocupa^40^.

A partir de estas características y de los objetivos planteados, se diseñó, en una segunda fase, el protocolo de entrevistas semiestructuradas para obtener información sobre la implementación del Consejo breve anti-alcohol llevado a cabo en las consultas de pediatría de atención primaria del Principado de Asturias. El guion contaba con cuatro áreas centrales: 1) contexto profesional; 2) percepción respecto a adicciones en la infancia/preadolescencia (conocer el posicionamiento de la o el profesional respecto al tema); 3) desarrollo de la intervención (consejo breve); 4) percepción respecto a la intervención (consejo breve). 

Finalmente, la muestra quedó conformada por 13 entrevistas (Tabla 1) al alcanzarse la saturación de la información, es decir, cuando en las entrevistas realizadas de forma sucesiva no apareció información nueva relevante para el objetivo de estudio^41^.

**Tabla 1:** Perfiles de las entrevistas realizadas. Principado de Asturias, España, 2022.

Código	Área Sanitaria	Sexo	Profesión	Edad	Fecha de realización
E4_médico	II Cangas de Narcea	Hombre	Pediatra	36-50	15/06/2022
E5_médica	III Avilés	Mujer	Pediatra	36-50	02/06/2022
E8_médico	IV Oviedo	Hombre	Pediatra	50-65	25/05/2022
E24_enfermera	V Gijón	Mujer	Enfermera	50-65	17/06/2022
E26_enfermero	VI Arriondas	Hombre	Enfermero	20-35	16/06/2022
E11_ médico	VI Arriondas	Hombre	Pediatra	36-50	08/06/2022
E13_médica	VII Mieres	Mujer	Pediatra	36-50	25/05/2022
E14_médica	VII Mieres	Mujer	Pediatra	50-65	19/05/2022
E17_médica	VIII Langreo	Mujer	Pediatra	20-35	24/05/2022
E16_enfermera	VIII Langreo	Mujer	Enfermera	50-65	03/06/2022
E2_médico	I Jarrio	Hombre	Pediatra	50-65	22/06/2020
E10_enfermera	V Gijón	Mujer	Enfermera	20-35	23/06/2022
E25_enfermera	V Gijón	Mujer	Enfermera	20-35	24/06/2022

Fuente: Elaboración propia a partir del trabajo de campo.

Las trece entrevistas se distribuyen de la siguiente manera en función de las variables de selección: una o dos entrevistas por área sanitaria, salvo en el área con más población que se realizaron tres; ocho con personal de pediatría y cinco con enfermería; ocho mujeres y cinco hombres en sintonía con la situación del sector; y una distribución prácticamente similar por edad con cuatro entrevistas a personas de 20 a 35 años, cuatro de 36 a 50 años y cinco a profesionales de 51 a 65 años. Para garantizar el anonimato y la confidencialidad de los datos se hizo uso de un sistema de pseudoanonimización con códigos que identifican a la entrevista y sus variables, pero no los datos personales que se corresponden con esta. 

El trabajo de campo se llevó a cabo entre los meses de mayo y junio de 2022 en los propios centros de salud, por preferencia de las propias personas entrevistadas. Esto ha facilitado la participación de los y las profesionales sanitarios, pero ha disminuido la duración media de las entrevistas que se sitúa en 22 minutos. Al ser una solicitud de evaluación de la propia Dirección General de Salud Pública de Asturias (España), esta investigación se ha adscrito a sus propios protocolos internos de actuación. Por tanto, no ha sido sometida a una revisión por parte del Comité de Ética de la Universidad de Oviedo. Ello no es óbice para que se hayan seguido estrictamente las normas del código deontológico de la Federación Española de Sociología y la Asociación Internacional de Sociología (ISA) al respecto de la confidencialidad, la privacidad, el anonimato y el derecho a la información de los y las participantes. Se han garantizado todas estas cuestiones durante todo el proceso de investigación y se han registrado a través de la firma, por duplicado y previa a la realización de la entrevista, mediante el consentimiento informado en el que se detalla específicamente el propósito del estudio, el procedimiento para la obtención de la información, los riesgos y beneficios de la participación, así como el derecho al abandono en cualquier momento. Las entrevistas fueron grabadas y transcritas para su posterior sistematización y codificación con el programa MAXqda.

La investigación y, en concreto, el análisis, se apoyan en la teoría fundamentada (*Grounded Theory* por sus términos en inglés), que permite la organización de las ideas que van emergiendo de los textos^41^ y, a partir de las cuales, la persona investigadora va identificando e integrando categorías inductivas de significado desde los propios datos^41^^,^^42^. En concreto, el proceso para crear categorías inductivas mediante la teoría fundamentada se inicia con la intencionalidad de extraer ideas susceptibles de construir categorías con base en preguntas de investigación. Para ello, se lleva a cabo una *codificación inicial* que identifica o da nombre a los conceptos que van apareciendo en el texto analizado. Tras esta identificación inicial, se transita de los conceptos iniciales a las categorías a través de una *codificación abierta* secuencial a lo largo del texto. Cuando se categorizan todos los conceptos presentes en los textos cualitativos. Tras este proceso, se reorganiza el sistema de categorías, mediante una *codificación axial*. Finalmente, se fija el sistema de categorías alrededor de una categoría central, la *codificación selectiva*, que va a ser el eje articulador del análisis^42^.

De este modo, el eje principal sobre el que se estructura el análisis es el grado de implementación y alcance de la intervención breve, mientras que las categorías axiales que emergen son las percepciones sobre la intervención y las adicciones reflejadas en los discursos de las y los profesionales encargados de su implementación. Estas categorías vertebran, posteriormente, la articulación de los códigos y subcódigos empleados en la codificación y análisis en este trabajo y dan origen a las tres líneas principales sobre las que se han estructurado los resultados: a)*“Grado de adecuación al protocolo y la guía en la intervención”;* b) “*Representaciones de las y los profesionales del servicio de pediatría respecto a la eficacia de la intervención”* y c)“*Percepciones de las y los profesionales del servicio de pediatría respecto a la cultura de consumo de alcohol en la infancia/preadolescencia*”. 

## RESULTADOS

Las entrevistas realizadas reflejan las interacciones que se producen entre las y los profesionales del servicio de pediatría y las niñas y los niños en edad pediátrica en torno a la iniciación en el consumo de alcohol, de acuerdo con lo que refleja el protocolo diseñado por la Dirección General de Salud Pública de Asturias, la Guía de Prevención y el propio Consejo breve anti-alcohol. No obstante, las narrativas recogidas van más allá y recogen aspectos contextuales, culturales y percepciones, recursos semánticos sobre los que se construyen los discursos y que sirven como ejes de análisis^30^^,^^32^^,^^33^.

### Grado de adecuación al protocolo y a la guía en la intervención (línea discursiva 1)

Lo primero que llamaba la atención en cuanto a la implementación del Consejo breve anti-alcohol es la laxitud en la realización de la intervención. Esto se constató desde el contacto previo a la entrevista, cuando se detectaron casos que desconocían la existencia del programa, o directamente se reconocía la no implementación. Más allá de estas respuestas minoritarias, la aproximación a la intervención entre quienes sí lo realizaban era dispar: junto a personas que indicaban que realizaban un seguimiento completamente ajustado al algoritmo, otros profesionales declararon que lo veían como una rutina más de los muchos protocolos que debían seguir en las revisiones pediátricas y había quienes se limitaban a anotar en la OMI-AP que habían hecho las preguntas. Por último, otras personas entrevistadas reconocían que lo aplicaban con mayor o menor minuciosidad en función del número de visitas y el tiempo disponible que tuvieran ese día:

“*A veces no me da tiempo* […]. *Claro, si tengo que prescindir de algo, suelo prescindir de eso*”. (E10_enfermera)

La mayor parte del personal sanitario se limitaba a realizar la primera pregunta de detección como una más del “Programa del niño sano” y la entrevista terminaba si el o la menor respondía negativamente. En algunas ocasiones, se limitaban a hacer alguna recomendación no protocolizada:

“*La adolescencia, bueno, es complicada para todos.* […] *Quizá me centraría en ponerle algún caso práctico y decir: ‘mira, he visto niñas de tu edad ingresadas por este por este motivo. Puede haber complicaciones graves y no solo a largo plazo*’”. (E17_médica)

Ninguna de las personas entrevistadas mencionó la continuación con las preguntas del cribado sobre las amistades, a pesar de la utilidad que la *Guía de prevención* de Astursalud afirma que tienen estas preguntas para identificar casos de consumo de alcohol en menores. La intervención finalizaba, de forma general, marcando la casilla correspondiente en la ficha de anamnesis del programa (la información aportada por el paciente para confeccionar su historial médico), aunque hay quienes reconocen que no siempre lo hacen. Por tanto, tampoco se continúa preguntando sobre las estrategias que utiliza el menor para mantenerse alejado del consumo de alcohol, pese a la recomendación de la propia *Guía de prevención*.

Aunque la ausencia de tiempo es señalada como la principal barrera, son varias las razones por las cuales las y los profesionales del servicio de pediatría no completan el protocolo, le dedican menos tiempo del recomendado o ni siquiera llegan a marcar la casilla de la ficha de anamnesis. Reconocen la importancia del Consejo breve anti-alcohol, pero lo consideran como una más de las políticas públicas de prevención que tienen que valorar. 

Gran parte de las y los profesionales del servicio de pediatría consideran que la prevención en materia de salud pública, aunque necesaria, es una tarea que excede en cierto modo sus funciones y que, en todo caso, pertenece a la enfermería. Ante la falta de tiempo, dentro de la revisión le dan prioridad a otros aspectos que consideran más propios de su profesión y relegan a un último plano lo que tiene que ver con mensajes preventivos y, particularmente, con el Consejo breve anti-alcohol.

*No sé, yo creo que igual lo del tiempo y cómo andamos a veces a carreras. Entonces no sé, priorizas a lo mejor* […] *auscultarlo, a mirarle la espalda, los pies, no sé qué, los dientes y tal y de eso a lo mejor, pues pasas. Si tienes un enfermero una enfermera competente, que la mayoría los tenemos, que sí que dedica mucho tiempo a la educación sanitaria y eso, pues sí.* (E5_médica)

Al mismo tiempo, las profesionales de enfermería tampoco sienten esta cuestión un aspecto muy propio de sus actuaciones a diferencia de otras recomendaciones como las del seguimiento de una dieta saludable. Argumentan que no se puede ofrecer una información detallada sobre el consumo temprano de alcohol, el consumo de tabaco, una alimentación saludable o sobre educación sexual en el tiempo que tienen para realizar la revisión pediátrica, por lo que deciden centrase en aquello que consideran más importante.

*Creo que yo no soy la persona indicada para dar esto porque yo soy la enfermera de pediatría, que tengo una serie de vacunas, que tengo además que dar una cantidad de información a los padres…* […] *Ahora tenemos esto sobre sexualidad. Ahora tengo esto sobre el tabaco. Les doy esto solo a los mayores.* […] ¿Sabes lo que te quiero decir? Porque, claro, nosotros no somos los que tenemos que formar en sexualidad, en el tabaco*, ni en el alcohol. Yo, desde luego, no estoy preparada.* (E24_enfermera)

Y, quienes sí que lo hacían, concluían que al tener que dar una cantidad ingente de mensajes preventivos se hacía muy difícil poder hacer otra cosa que pasar de puntillas por ellos.

*También te digo… Sí, tengo algo de experiencia, pero si voy con prisa,* […] *no me da tiempo para hablar con ellos* […] *eso requiere un poco de tiempo para hablar* […] *No puedo sacarles el tema directamente tal cual nada más entran* […] *A veces no me da tiempo* […]. *Claro, si tengo que prescindir de algo, suelo prescindir de eso, sí.* (E10_enfermera)

Más allá de la premura de tiempo, se ofrecían otras explicaciones que, en ocasiones, respondían más a ideas preconcebidas o sesgos cognitivos y que explicaban cómo se llevaba a cabo la intervención^33^^,^^34^. Se consideraba que las y los pacientes de 10 años eran demasiado infantiles, apreciaban un riesgo realmente bajo y no encontraban procedente realizar el consejo o decidían que había otras intervenciones prioritarias.

*Vimos el programa y bueno, dijimos “bueno, vale, hay que hacerlo”* […] *Una información para padres y otra para niños. Y luego* […] *cuando ya le hacía la revisión tal y entonces en los consejos un poco hablando “bueno y acuérdate que, o ten en cuenta que el alcohol es muy malo, que no debes tal, tal, vale”. Lo hacía a los diez años y a los trece. Lo hice así poco tiempo porque a los 10 años* […] *me miraban los niños* […] *y los padres con una cara, que digo: “bueno, ¿por qué le tengo que estar hablando del alcohol con diez años?” Me parecía absurdo. Y entonces empecé a hacerlo solo a los trece. Y a los trece lo hago. No siempre me acuerdo*. (E13_médica)

En la revisión de 13 años, en cambio, sí se apreciaba una mayor necesidad de aplicar el Consejo breve anti-alcohol, pues se acercaban a la adolescencia, la edad considerada crítica por todas las personas entrevistadas. Empiezan a salir con amigas y amigos lejos del control parental y es apreciable ya el desarrollo físico y psíquico, especialmente, en el caso de las niñas^9^^,^^12^.

*Hombre, un niño a los diez años está más infantilizado. Y sobre todo si es un niño respecto a las niñas, ¿no? Que siempre están un poco más avanzadas, o sea, que vemos a los diez años son casi… Son niños, mentalmente, en cambio ya a los trece, sobre todo las niñas, son muy maduras y claro que hay diferencia con el tema*. (E2_médico).

Sorprende que algunas personas tuvieran una percepción de menor riesgo, dependiendo de la ubicación de la consulta de atención primaria. En general, existe una consideración de menor riesgo en aquellas con perfiles familiares de nivel socioeconómico alto, lo que puede dar lugar a hipótesis erróneas que dificulten la detección temprana.

*Pues aquí poco frecuente, la verdad, pero por cuestión de la zona, ¿eh? Esta es una zona de nivel socioeconómico medio-alto* […] *y poco de eso se ve, la verdad*. (E10_enfermera)

En cualquier caso, muchas personas consideraban que carecían de la formación adecuada. Y no se referían solo a la metodología o las herramientas necesarias para la implementación de la técnica, sino que detectaban grandes carencias en las habilidades sociales necesarias para afrontar la interacción con las familias y con las y los menores. En el caso del Consejo breve anti-alcohol, este déficit parecía relevante en relación con la falta de formación en habilidades sociales y de comunicación aludidas^7^^,^^26^.

*Claramente porque todavía no tengo la experiencia como para meterme en esos meollos. Igual que te digo que en prevención de conductas de riesgos sexuales, ¿eh? Quiero decir, que ahí también tengo muchos déficits. No sabría cómo abordarlo realmente* [...] *las veo complejas porque me falta muchas herramientas a nivel de hablar, de poder comunicarme con ellos, efectivamente*. (E17_médica)

A pesar de la laxitud en la implementación del Consejo breve anti-alcohol por parte de algunas y algunos profesionales, aquellas personas que dedicaban más tiempo al protocolo parecía que seguían con bastante fidelidad las recomendaciones para el trabajo con menores en cuanto a la no utilización de lenguaje alarmista, trasladar un mensaje claro de la no existencia de un consumo sin riesgo o potenciar las habilidades relacionadas con la presión del grupo de iguales. 

### Percepciones de las y los profesionales del servicio de pediatría respecto al alcance de la intervención (línea discursiva 2)

A pesar de la complejidad de la intervención, la relevancia del Consejo breve anti-alcohol estaba fuera de toda discusión^26^^,^^30^. Se consideraba que cualquier actuación que incidía en la prevención de riesgos relacionados con la salud debía realizarse. Las narrativas eran menos optimistas, sin embargo, en cuanto a la efectividad del programa o, al menos, no alcanzaban a ver su impacto. Las razones esgrimidas eran varias. En primer lugar, la dificultad para valorar una intervención difícil de cuantificar. Para explicarlo, acudían a la comparación con otras de las actuaciones que realizaban en consulta. Así era fácil ver el éxito de otras medidas encaminadas a mejorar la salud de las y los pacientes pediátricos, por ejemplo, observando si ganaban talla o peso. En el caso del Consejo breve anti-alcohol, no se podía conocer la efectividad -aún en el caso de que algún paciente o sus familias requirieran la intervención-, ya que las adicciones son asuntos socialmente indeseables y cuyas consecuencias se ocultan.

*Hombre, ¿yo cómo lo percibo? Pues mira, es muy difícil, porque, por ejemplo, tú me puedes preguntar* […] *sobre un programa de obesidad. Entonces, ¿cómo ves la efectividad del consejo? La dieta de* [Inaudible] *fácil* […] *porque le vas pesando y ves si gana peso o si pierde. Pero este tema, ¿cómo lo valoras y cómo tal? Es muy difícil, o sea que es muy difícil de valorar, de seguir y de todo. Claro, ¿cómo se mide eso?* (E2_médico).

Por otra parte, resultaba también complejo realizar un seguimiento en el tiempo, ya que el programa estaba destinado a pacientes pediátricos al borde de abandonar tal condición (especialmente en la consulta de 13 años) por lo que en muchas ocasiones no volvían a tener contacto con los niños ni con sus familias.

*Tú date cuenta de que vemos pacientes hasta los catorce años. Si bien es cierto que hasta esa edad el contacto con el alcohol puede ser ocasional. Cuando se hace mucho más agudo (es) a partir yo creo que, de los dieciséis, que es cuando entran en una adolescencia mucho más profunda y dificultosa.* (E17_médica)

En tercer lugar, la percepción que tenían las y los profesionales es que niñas, niños y adolescentes se informaban a través de otros canales, fundamentalmente grupo de pares, redes sociales y, en menor medida, familias. En este sentido, las respuestas de las personas entrevistadas contradecían una de las premisas halladas en la bibliografía que situaba a las y los profesionales del servicio de pediatría como referentes y fuente más apropiada para recibir mejor información y más objetiva sobre drogas^31^. Este hecho se constata a partir de las escasas consultas sobre adicciones que las y los profesionales sanitarios indicaban recibir.

*Que yo conozca, no. Que yo conozca no, porque los vemos hasta justo cuando cumplen los catorce años y no he tenido ningún caso* […] *me imagino que habrán cogido sus borracheras y demás, pero como adicción al alcohol como tal en la historia clínica… no*. (E8_médico)

*No, no* […] *yo creo que demanda de una consulta de ese tipo nunca la tuve*. (E14_médica)

Solo algunas o algunos profesionales consideraban que llegaban a alcanzar una cierta confianza, quienes llevaban años ejerciendo en el mismo centro de salud. Esto se relacionaba de forma directa con una problemática que afecta, fundamentalmente, a las y los profesionales más jóvenes: una precariedad laboral caracterizada por la temporalidad y la elevada rotación entre distintos centros de salud que. Según su propia percepción, era un aspecto que dificultaba la complicidad con niñas y niños al no tener a una persona de referencia, sino a varias a lo largo de su infancia y preadolescencia. No obstante, esta precariedad laboral no parecía ser considerada como una barrera para llevar a cabo la intervención y efectivizar una política pública.

*Yo creo que esto ya es un problema estructural, que probablemente sería más efectivo e hicieran más caso si la continuidad del personal de atención primaria fuera mayor. Si son niños que llegan a los diez* [o] *a los trece años, pero me conocen desde que han nacido (desde los tres, desde los cuatro) y tienen confianza conmigo, soy una figura de referencia y la relación es buena, creo que el consejo puede calar mucho más que si cada vez que vienen les atiende una persona diferente* […] *llegan a la puerta y no me conocen de nada* […] *después les estoy preguntando si han consumido alcohol o si no....* (E26_enfermero).

De este modo, los factores mencionados, junto con las percepciones respecto a las adicciones en la infancia-preadolescencia -sobre las que se profundiza en el siguiente apartado- van a condicionar las maneras de abordar la intervención y, en especial, la forma diferenciada de proceder a los 10 y a los 13 años. 

### Percepciones de las y los profesionales del servicio de pediatría respecto a la cultura de consumo de alcohol en la infancia/preadolescencia (línea discursiva 3)

Las y los profesionales del servicio de pediatría verbalizaban en sus discursos que el consumo de alcohol entre los menores se iniciaba cada vez más temprano. Por esta razón, existía una conciencia generalizada de que es un problema que debe ser atajado cuanto antes, debido a los daños que causa en personas física y psíquicamente en desarrollo, pero también en sus familias y en la sociedad^7^.

*Yo creo o percibo que sí. No sé desde cuándo, pero yo creo que, además, de forma más precoz están en contacto con las sustancias, con las sustancias de las que luego pueden abusar. No solamente el alcohol sino también el tabaco* […] *Y los ves por la calle y… bueno, sabes que pueden llegar a beber*”. (E4_médico)

Las personas entrevistadas se mostraban partidarias de la tolerancia cero con el alcohol y eran especialmente críticos con la doble moral existente a nivel institucional. Varias personas entrevistadas criticaban la utilización de una doble moral en la que coexistían programas de prevención temprana como el Consejo breve anti-alcohol con la utilización de mensajes peligrosos de consumo responsable, diferenciación entre bebidas alcohólicas según su graduación o, la realización de promociones desde los ámbitos institucionales^9^^,^^13^.

*Las autoridades tampoco lo controlan tanto* […] *favorecen la fiesta de la cerveza, la fiesta de la sidra, la fiesta de eso y todas esas cosas como que no…* […] *como que es una contradicción de muchas cosas a la vez* […] *si se lo pones tan a mano… Lo cual no quiere decir que se lo estés poniendo tú a mano, pero evidentemente los adolescentes que todo lo quieren probar* […] *pues si tú le facilitas muchas fiestas, pues ellos van a ir y, sobre todo, si no se les controla.* (E24_enfermera)

Pero, más allá de las instituciones, se reconocía que el problema estaba fuertemente enraizado en las familias. Existía una alta tolerancia con el alcohol, a diferencia de lo que ocurría con otras sustancias adictivas como el tabaco, de las que se tenía una percepción de mayor nocividad.

*Tenemos muy normalizado en los padres, decir: “¿quedamos a tomar una cerveza? ¿Vamos a tomar unos culetes? ¿Tomamos un vino?”. Ya. Eso lo decimos como una cosa normal* […] *Ellos no se van a enterar, pero con diez, trece años, eso lo ven como algo normal* [...] *Sin embargo, ven peor el decir: “¿salimos a echar un pitín?”* […] *Sí, no vamos a hablar de borracheras, ni bebida blanca, ni demás… pero el hecho de que por la mañana tus padres se van a tomar un vino, pues es la cosa más normal del mundo, un vino o dos o los que tomes.* (E16_enfermera)

También resulta muy complejo sustraerse a la presión social que ejercen los medios de comunicación, las redes sociales (de forma más reciente) y, especialmente, el grupo de iguales^43^^,^^44^^,^^45^. La coincidencia era clara respecto a que solo aquellos o aquellas jóvenes con un mayor grado de madurez eran capaces de resistirla.

*Los adolescentes se guían por iguales. Entonces, independientemente de cuál sea el gatillo que haya hecho que uno de ellos beba, que puede ser por múltiples causas, los demás, yo creo que, si no tienen una personalidad muy, muy clara, que pocos la tienen a la edad de los trece años, van a ir detrás porque no saben diferenciar lo bueno de lo malo. Todavía prima, bueno, pues el estar en la cabeza de la ola guay. Y, no sé, la verdad es que… tengo mis dudas de dónde va a acabar esto, pero mucho le echo la culpa a las redes sociales, ¿eh? Cuidado.* (E17_médica)

Todas las personas entrevistadas expresaban este sentir, si bien las de menor edad se mostraban más comprensivas con la iniciación en el alcohol, ya que tenían más recientes sus propias experiencias y trataban de explotar esta menor diferencia de edad para legitimar su discurso.

*Yo intento tener una relación con el adolescente un poco más de iguales y decirle que, a ver, que yo entiendo que van a empezar a salir, que van a empezar a probarlo, pero que conozcan también los límites y los riesgos que puede conllevar el pasarse de ciertos límites.* (E10_enfermera).

La percepción era que, aunque el inicio del consumo sea cada vez más temprano, la edad en la que se evidenciaban los problemas y consultas en torno al alcohol era posterior a la edad pediátrica. Y así parecían reforzarlo las encuestas que situaban esa edad a los 14 años, justo cuando las y los profesionales del servicio de pediatría dejaban de verlos^26^. Resulta paradójica, por tanto, la concienciación sobre el problema junto a una ausencia casi total de la constatación de casos en su entorno^32^. Cuando se preguntaba a las y los profesionales del servicio de pediatría por las situaciones que atendían en atención primaria, raramente aparecían consultas sobre adicciones por parte de las familias o de los propios niños y niñas. 

## DISCUSIÓN

Las organizaciones sanitarias mundiales han subrayado la importancia de las intervenciones breves y del seguimiento continuado para asegurar su efectividad, especialmente en la población joven^6^. Sin embargo, la implementación efectiva de estas intervenciones requiere un enfoque sistemático y apoyo institucional que, según nuestros hallazgos, está actualmente limitado.

El artículo ha puesto de relieve cómo la ejecución del Consejo breve anti-alcohol en la práctica pediátrica muestra una notable variabilidad, tanto en el grado de aplicación como en las percepciones de su efectividad. La heterogeneidad observada no es única en el contexto español, ya que resulta consistente con los hallazgos de la literatura sobre la cuestión^5^, en la que se han hallado variaciones significativas en el uso de intervenciones breves relacionadas con el alcohol entre diferentes países de ingresos bajos y medianos. Estudios internacionales han resaltado, asimismo, la falta de estandarización y el reto de integrar estas intervenciones en la rutina clínica diaria^23^^,^^24^, documentando desafíos similares en otros países europeos en los que las barreras incluyen tanto la falta de formación, como la percepción de que las intervenciones preventivas sobre el consumo de alcohol no son prioritarias en el entorno pediátrico​.

La dificultad para valorar la efectividad del Consejo breve anti-alcohol es otro aspecto crítico y es considerado un obstáculo significativo en nuestra investigación por las y los profesionales entrevistados. Además, la falta de herramientas estandarizadas para evaluar los cambios en los patrones de consumo de alcohol entre adolescentes complica aún más el proceso de medición. Mientras que, en otras áreas de la salud pediátrica, como la de nutrición, los resultados pueden ser medidos de manera tangible a partir de indicadores morfométricos, la efectividad del Consejo breve anti-alcohol es más difícil de cuantificar, ya que el consumo de alcohol se manifiesta de manera más sutil y a largo plazo. Uno de los principales desafíos para la medición radica en la naturaleza compleja y multifactorial del consumo de alcohol, que está influenciado por factores sociales, psicológicos y culturales. Este hallazgo se alinea con estudios previos^25^ que han mostrado que los resultados de las intervenciones breves en la prevención del consumo de alcohol son difíciles de cuantificar, debido a la naturaleza socialmente indeseable de las adicciones, al ocultamiento de sus consecuencias y a la falta de medidas tangibles y de un seguimiento prolongado que dificulta la evaluación del impacto de estas intervenciones y limita la precisión de los datos obtenidos durante las consultas. Además, las y los profesionales de la salud aluden a la desconexión entre la atención pediátrica y la adolescencia tardía, cuando los problemas relacionados con el alcohol son más evidentes, lo que socava la capacidad para medir los resultados a largo plazo. Como pone de relieve, también, nuestra investigación: las personas dejan de ser atendidas por los servicios de pediatría al alcanzar la adolescencia tardía, justo cuando los problemas relacionados con el alcohol tienden a manifestarse con mayor claridad.

Por otra parte, nuestro artículo evidencia la importancia de un marco institucional sólido para abordar las disparidades en la implementación de intervenciones preventivas, abundando en los hallazgos de la literatura sobre la cuestión. En el ámbito internacional las organizaciones sanitarias recomiendan la integración de programas de intervención breve como parte de un enfoque de salud pública más amplio^6^ y, a nivel nacional, también se ha confirmado la importancia del apoyo hacia las estrategias psicoeducativas desde las instituciones^8^. En el contexto de nuestra investigación, gran parte de las y los profesionales del servicio de pediatría no perciben la intervención anti-alcohol como una parte integral de sus responsabilidades principales, a pesar de que la intervención es considerada relevante para la prevención de riesgos relacionados con el alcohol. Esta percepción se ve exacerbada tanto por la falta de formación en habilidades sociales y comunicativas, esenciales para abordar de manera efectiva temas de prevención con menores y sus familias, como por la falta de apoyo administrativo, que obstaculiza de manera significativa la aplicación efectiva del Consejo breve anti-alcohol.

El contexto social y cultural también juega un papel importante en la percepción y la efectividad de las intervenciones preventivas. Uno de los roles claves en el desarrollo de los y las menores que destaca la literatura internacional para el personal de pediatría es el de cuidar y educar, más allá del papel de proveedores de salud, orientando en aspectos clínicos, pero también en la promoción de hábitos de vida saludables. Estudios pioneros en la teoría del aprendizaje social^20^, pero también otros más recientes^25^ han puesto de manifiesto que las y los adolescentes son particularmente susceptibles a las influencias del entorno, observando y replicando los comportamientos de figuras de referencia, como familiares, amigos y figuras públicas. De este modo, las y los profesionales del servicio de pediatría tienen la oportunidad de intervenir de manera proactiva, no solo brindando consejos preventivos directos, sino también como agentes educativos, modelando comportamientos saludables y fomentando un entorno que desaliente el consumo de sustancias nocivas. Al proporcionar a los progenitores las herramientas y conocimientos necesarios para promover un ambiente libre de alcohol y otras drogas, se potencia el aprendizaje social positivo y se reduce el riesgo de que los y las menores adopten patrones de comportamiento perjudiciales, al tiempo que aumenta en las y los adolescentes el nivel de autoeficacia, es decir, su percepción de que son capaces de controlar el consumo. 

Un enfoque integral de este tipo no solo se centra en la salud física, sino también en el bienestar emocional y social de las y los menores, considerando que el aprendizaje de comportamientos saludables es un proceso continuo que se extiende más allá del entorno clínico, abarcando el hogar, la escuela y las comunidades. En el contexto español, la literatura ha identificado a las y los profesionales de pediatría como fuentes confiables de información^8^, sin embargo, nuestros hallazgos muestran que las y los profesionales consideraban que las y los adolescentes preferían obtener información de sus pares, redes sociales y, en menor medida, de sus familias. Nuestra investigación, por tanto, se sitúa en línea con otros estudios que, en el ámbito internacional señalan que las influencias de los pares y las redes sociales son factores determinantes en el comportamiento relacionado con el alcohol entre las y los jóvenes, como fuente de influencia en el comportamiento, de acuerdo con modelos de comportamiento como la teoría del aprendizaje social^20^^,^^25^^,^^43^^,^^44^^,^^45^.

Esta disonancia con la literatura parece destacar la necesidad de adaptar las intervenciones preventivas a las realidades locales y a las características del sistema de salud en España. La variabilidad en la implementación observada en el artículo sugiere que, si bien los modelos generales pueden ofrecer una base útil, las intervenciones deben ser contextualizadas para ser efectivas en diferentes entornos nacionales y culturales.

## CONCLUSIONES

La aplicación del Consejo breve anti-alcohol en edad pediátrica se ha manifestado inconsistente. Los resultados indican la priorización de las intervenciones clínicas sobre las preventivas, por lo que las personas entrevistadas realizan esta acción, ya breve en su diseño, de forma mecánica. De esta forma, las intervenciones se limitan a una conversación superficial y a su correspondiente registro en los soportes digitales habilitados, no alcanzando, según la evaluación realizada, el impacto y el alcance deseados. 

 El artículo destaca la necesidad de un enfoque más sistemático y de un apoyo institucional para la implementación de intervenciones preventivas. Se subraya la necesidad de un enfoque integrado de salud pública para afrontar el consumo de alcohol entre adolescentes, mejorar la consistencia de las intervenciones, maximizar su efectividad y superar las barreras que limitan su implementación. De igual forma, la percepción de que el análisis longitudinal es limitado contribuye a una baja eficacia en la prevención del consumo de alcohol. En nuestro estudio se ha puesto de relieve que el diseño de estrategias que permitan un seguimiento más efectivo de las y los adolescentes, más allá de la edad pediátrica, podrían asegurar una intervención continua y más efectiva en la prevención del consumo de alcohol.

A estas barreras estructurales se suma la percepción de las y los profesionales del servicio de pediatría sobre su papel en la prevención del consumo de alcohol entre adolescentes y la dificultad de establecer una relación de confianza en una intervención que habitualmente se realiza, a pesar de lo que indica el protocolo, con los progenitores presentes, por lo que se introducen sesgos en la respuesta de las experiencias con el alcohol de las y los adolescentes. Quizás por ello, aunque el personal de pediatría es reconocido como la fuente o vía de información más apropiada y objetiva, habitualmente se emplean otros canales como el grupo de pares, las redes sociales o, en menor medida, las familias. Este es un hecho ratificado por las escasas consultas sobre adicciones que las y los profesionales sanitarios refieren y que podría estar relacionado, asimismo, con la contradicción existente entre un hábito de consumo muy arraigado en nuestro país (y en Asturias en particular), por un lado, y la naturaleza socialmente indeseable de las adicciones, por otro.

Algunas de estas dificultades, descritas en las entrevistas, están relacionadas con la ausencia de formación o la no adecuación de esta, lo que se traduce en falta de herramientas prácticas e insuficientes habilidades para afrontar las situaciones que se dan en consulta, pero también con los sesgos cognitivos del personal sanitario que ha de proporcionar el consejo breve. De ahí que, de forma específica, se sugiera la creación de programas de aprendizaje continuos en habilidades sociales y comunicativas para las y los profesionales del servicio de pediatría, adaptados a la edad y el sexo de las y los pacientes en edad pediátrica, así como a las posibles especificidades locales.

Por último, cabe destacar que la bibliografía científica concluye que existen pautas y actitudes de consumo de alcohol en adolescentes diferenciadas por sexo. Sin embargo, no ha sido posible verificar este extremo en el curso de esta evaluación, ya que no se puede constatar la existencia de adicciones de menores, especialmente en la primera fase del cribado, de diez a trece años. Este es un rango de edad en el que las familias se muestran remisas a hablar sobre la cuestión y en el que el propio personal de pediatría aprecia un riesgo bajo, por lo que no considera procedente realizar el Consejo breve o entiende que tiene baja prioridad con relación a otras intervenciones.
